# Accuracy of Three-Dimensional Computer-Aided Implant Surgical Guides: A Prospective In Vivo Study of the Impact of Template Design

**DOI:** 10.3390/dj13040150

**Published:** 2025-03-29

**Authors:** Noel Vartan, Lotta Gath, Manuel Olmos, Konstantin Plewe, Christoph Vogl, Marco Rainer Kesting, Manfred Wichmann, Ragai Edward Matta, Mayte Buchbender

**Affiliations:** 1 Department of Oral and Maxillofacial Surgery, Friedrich-Alexander-Universität Erlangen-Nürnberg, Glückstrasse 11, 91054 Erlangen, Germany; noel.vartan@uk-erlangen.de (N.V.); lotta.gath@uk-erlangen.de (L.G.); manuel.olmos@uk-erlangen.de (M.O.); marco.kesting@uk-erlangen.de (M.R.K.); 2 Department of Prosthodontics, University Hospital Erlangen of Friedrich-Alexander Universität Erlangen-Nürnberg, Glückstrasse 11, 91054 Erlangen, Germany; konstantin.plewe@uk-erlangen.de (K.P.); manfred.wichmann@uk-erlangen.de (M.W.); ragai.matta@uk-erlangen.de (R.E.M.)

**Keywords:** guided implant surgery, surgical guide template, CAD/CAM surgical guide, dental implant

## Abstract

**Background**: Digital planning and the use of a static surgical guide for implant placement provide predictability and safety for patients and practitioners. The aim of this study was to investigate differences in the accuracy and fit of long and short guides. **Methods**: In patients with at least one missing tooth, long (supported by the entire dental arch) and short templates (supported by two teeth, mesial and distal) were compared via intraoral scans and the superimposition of the STL files of the initial planning and the actual position in the patient’s mouth along the X-, Y- and Z-axes. Furthermore, this study evaluated the conditions (e.g., mouth opening, the implant position) under which fully guided implantation can be realized. **Results**: The largest deviation was observed in the Z-axis, although this deviation was not as high for the short templates (0.2275 mm) as it was for the long templates (0.4007 mm). With respect to the 3D deviation (dXYZ), the average deviation from the mean value was 0.2953 mm for the short guides and 0.4360 mm for the long guides (*p* = 0.002). The effect size (Cohen’s d) was 0.709, which was between the medium (0.50) and large effect sizes (0.80). The shorter templates showed a smaller deviation from the actual plan by 80%. With a mouth opening ≥50 mm, fully guided surgery can be performed in the molar region. In the premolar region, the lower limit was 32 mm. **Conclusions**: The 3D accuracy was significantly higher for the shorter template, which could therefore be favored.

## 1. Introduction

Dental implantation is one of the most common procedures in dental surgery. Accordingly, the global market for implants was expected to be worth approximately USD 13 billion in 2023 [1]. Dental implantation, the avoidance of complications and the longevity of dental implants are highly important for patients, but this procedure poses challenges for surgeons. For decades, implants were placed in a freehand-guided (FH) manner; currently, in the age of digitalization, digitally planned and printed surgical guides/templates are becoming more widespread [2,3]. In the past, practitioners without a defined surgical guide had to react and pay attention to aspects such as the axial alignment and depth of the implant placement intraoperatively during implantation; however, currently, the exact position of the implant can be predicted, especially from a prosthetic perspective (i.e., backwards planning), and planning can be completed during the operation. Thus, the implant placement becomes more accurate, especially with a higher degree of guidance while drilling the implant [4,5,6]. Three different ways of guided surgery implant insertion are available—using the static surgical template to guide the drills and placing the implant through this (full guided) [7], just using the template guide while drilling (half-guided) or just performing the pilot drill through the guide. A systematic review revealed that the fully guided protocol demonstrated a higher level of accuracy compared to the pilot-guided protocol, but without significance when compared to the half-guided protocol. Thus, several studies agree that the full guided protocol is the gold standard [8].

Many companies are using different ways to guide drills during the guided surgery [9], while the literature is inconsistent regarding which way might be better than others [10,11]. Therefore, in an in vitro study, 140 implants were placed in identical mandible replicas through seven different drill guide systems. They evaluated the overall angular deviation of the implants using either drill handle, drill-body guidance (systems where the key is attached to the drill), or combinations thereof. They concluded that sleeve heights ≤4 mm correlated with higher angle deviations from the planned implant position, and that the angular deviation ranged significant between the drill handles and the drill-body guidance systems (0.88 ± 0.41° vs. 3.97 ± 2.01°). Thus, systems with drill handles reached the highest accuracy [9].

In oral and maxillofacial surgery, CAD/CAM (Computer-Aided Design/Computer-Aided Manufacturing) and navigation-assisted systems offer significant advantages as they enable exceptional precision and the individualization of procedures. By using CAD/CAM technologies, surgeons can create detailed 3D models of the jawbone and surrounding tissues to develop precise surgical plans. This is particularly beneficial for complex procedures such as the reconstruction of facial defects. Custom-made implants and surgical aids based on the models improve the accuracy of fit and reduce the risk of maladjustment. Navigation technology, in turn, makes it possible to track the exact positions of instruments in real time during surgery. These technologies help to minimize risks, speed up healing processes and significantly improve aesthetic results, which have been proven by studies in respective systematic reviews [12,13]. Another systematic review dealt with the question of whether intraoperative navigation might even improve the margin status in advanced malignancies of the anterior craniofacial area. They themselves analyzed 70 patients with and without navigation, and assessed the margin status. This demonstrated a significantly lower rate of positive margins in the navigation group, particularly in pT4 tumors and recurrences, whereby the operation time did not differ. Further, the systematic analysis of a total of 209 patients showed a significantly better rate of negative margins. For this reason, the authors would recommend navigation in particular for advanced or complex tumors [14]. However, the navigation can be used not only for facial reconstructions or tumor patients, but also for orthognathic surgery. In a systematic review, the authors analyzed 12 publications, of which 3 were randomized clinical trials and 9 were observational studies. All studies reported a clinically acceptable outcome with a 2 mm difference between the planned and postoperative situations. Navigation was also shown to be significantly more accurate at up to 0.6 mm compared to the conventional surgical approach [15].

In digital implant planning, hard and soft tissues are evaluated during the analysis and planning of the implant position; the subsequent prosthetic restoration is simulated, and the finished drilling template transfers this plan to the patient’s oral situation. The advantages of digital planning are as follows: predictability, the protection of neighboring anatomical structures, the perfect prosthetic position of the implant, and a reduced operating time for the patient [16,17,18,19]. However, guided surgery also has several disadvantages, as follows: increased time is needed for planning; the template may not fit the patient appropriately; and the template or so-called full-guided surgery may not be feasible due to a restricted mouth opening [20,21].

In such situations, the surgeon only has the option of resorting to FH surgery or, under certain circumstances, using the fabricated template for pilot drilling (i.e., half-guided) or as a positioning template without the corresponding guided accessories (i.e., the drill and guidance system). While little can be changed in terms of the patient’s mouth opening, it is not surprising that there has been an increasing amount of research related to designing and manufacturing surgical guides. This is because these guides can either be individually designed by the software user (e.g., coDiagnostiX 10.9 Dental Wings) or a central production site can be commissioned to design and manufacture the templates [22]. The templates should always be designed in accordance with standard requirements (stability, good and tight fit and sufficient support area while drilling).

In terms of manufacturing methods, additive manufacturing techniques (3D printers) and subtractive techniques (milling) are in a clearly clinically acceptable range in terms of their fit [23]. Moreover, there is little or no difference in the design of the guides examined to date, as they always cover all teeth in the jaw that are still in situ for support [24]. The drilling template design over the entire dental arch appears to be intuitively the most advantageous, especially in operations in which several implants are placed and in free-end gaps, as the large support surface also offers a high level of stability.

However, clinical applications have shown that these designs can often lead to the swinging of the template intraoperatively, and thus to an inaccurate fit of the guides. This results in undesirable deviations in the final implant position. We hypothesize that the resulting leverage effect is weaker and that the accuracy of fit in the mouth is higher due to a shorter template span. Additionally, there are currently few studies that have compared the fit and accuracy of different spans with digitally planned surgical guides. Therefore, the current study performed this comparison.

## 2. Materials and Methods

This prospective clinical trial included patients who presented to the Department of Oral and Maxillofacial Surgery at the University of Erlangen-Nürnberg in 2024 due to the need for dental implant placement. During the planning process, each implant was planned and placed with the help of the digital planning software coDiagnostiX (Version 10.9, Dental Wings, Straumann Group), which represents the standard procedure at the department. Additionally, digital volume tomography and an intraoral scan (Trios 4; 3 Shape; Copenhagen, Denmark) were taken from each patient.

The CBCT scans yield files in the standard medical format DICOM (Digital Imaging and Communications in Medicine). In contrast, intraoral scans are here saved as standard tessellation language (STL) files. Two templates were compared in the course of the investigation, and both were assigned to each patient: a long template that rests on all remaining teeth, and a short template that rests on two teeth mesial and distal to the planned implant position. Patients with a distal extension of the edentulous space were not included in this study. Moreover, patients with adjacent mobile teeth or generally impaired periodontal status were excluded.

The inclusion criteria were as follows: at least one missing tooth and need for a dental implant. Ethical approval (registration No. 24-338-Br) for this study was obtained from the ethics committee of the medical faculty of Friedrich-Alexander University Erlangen-Nürnberg, Germany, and the guidelines of the Declaration of Helsinki were followed. This study was also registered within the German Clinical Trials Register (No. DRKS00036228). Adult patients of all ages and sexes were included in the study after providing informed consent. Furthermore, each patient was free to withdraw from the study at any time without providing reasons. If one of the potential test subjects did not agree with the performance of the study or did not wish to participate in the study, they did not suffer any disadvantage with respect to further treatment.

### 2.1. Design and Printing of Two Different Surgical Guides

**Figure 1:**
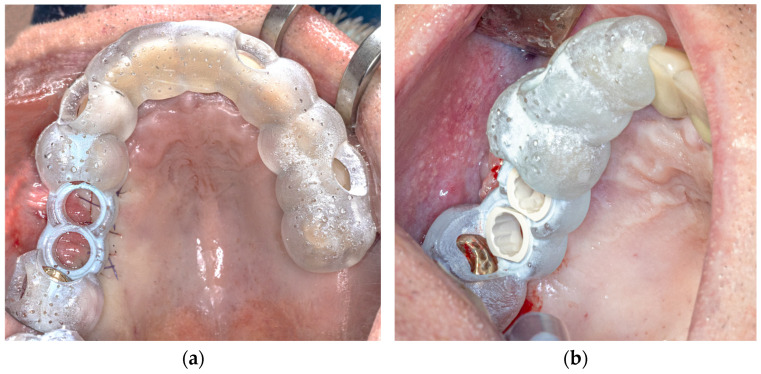
(**a**) Long surgical guide template; (**b**) short surgical guide template.

For the configuration of the longer template, all the remaining teeth of the jaw were selected as the supporting surface (Figure 1a). The support of the shorter template was limited to four teeth, which were designed symmetrically with two teeth mesial and distal to the planned implant position, if possible. If there was only one tooth distal to the implant position, this tooth and the three teeth mesial to the implant position were included, and vice versa (Figure 1b).

The apical extension of the templates always ended in the lower third of the clinical tooth crown. The contact surface was therefore exclusively dental and never reached the gingiva. The offset was then set to 0.2 mm, and the wall thickness was set to 0.3 mm. Finally, viewing windows were evenly added to the templates to permit us to check their fit in the best possible way.

The two surgical guides were prepared for 3D printing via Netfabb Standard 2020 software (Autodesk; San Rafael, CA, USA) and then 3D printed via a Sheraprint 20 3D printer (SHERA; Hamm, Germany), which uses digital light processing (DLP) technology.

### 2.2. Surgical Procedure

All surgical procedures were performed under local anesthesia (Ultracain DS; adrenaline 1:200,000; Sanofi-Aventis GmbH, Frankfurt, Germany). All the implant insertions were performed by one experienced oral surgeon at the Department of Oral and Maxillofacial Surgery. Each implant healed in a pattern of subgingival healing, and the wounds were stitched with Vicryl^®®^ 5-0 Rapide (Ethicon GmbH & Co KG, Norderstedt, Germany). Patients were instructed in terms of nutrition and postoperative events, such as bleeding or pain and swelling; moreover, they were administered pain killers if needed, and follow-up appointments were scheduled.

### 2.3. Outcomes

The primary outcome was defined as the in vivo accuracy of both (long and short) 3D-printed implant templates in terms of the 3D deviation of the implant drilling position in the patient compared with digital planning.

The secondary outcomes included the conditions (e.g., mouth opening, implant position) under which a fully guided implantation can be realized, as well as preferences for a certain type of template design, which were identified based on the fully guided implantations performed and the implant position. These outcomes were evaluated by the surgeon using the following parameters: implant position; interincisal distance; template used; lower deviation dXYZ in the analysis; and the feasibility of fully guided implantation.

### 2.4. Digital Examination

The 3D evaluation of the collected data was carried out via the CAD analysis software ZEISS INSPECT Optical 3D, Version 6.3 (Carl Zeiss AG, Oberkochen, Germany).

**Figure 2:**
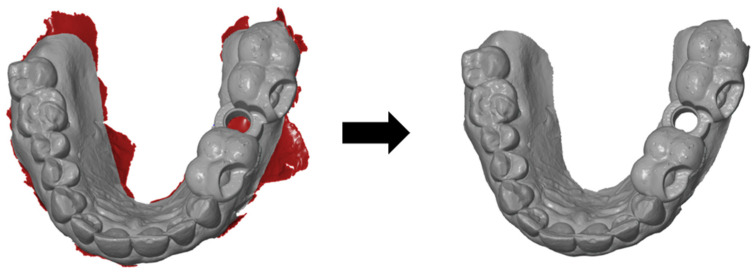
Removal of artifacts on an intraoral scan with a tried-in template.

The basic aim of this analysis was to determine the actual three-dimensional deviation of the short and long templates from the respective planned implant position. To standardize all of the scans, the movable mucosa in the vestibule had to be removed initially, as this does not allow clear results due to its displaceability. Owing to the lack of space in the molar region, the scans with the fitted templates often resulted in artefacts that had to be removed afterwards (Figure 2). The cylindrical opening at the implant position was also always cut free, as this opening was often falsely filled by the intraoral scanner.

After this initial processing, four STL files per patient were obtained; these files included the long and short template scanned on one patient and twice the optimal positions of the respective template type generated by the implant planning software.

**Figure 3:**
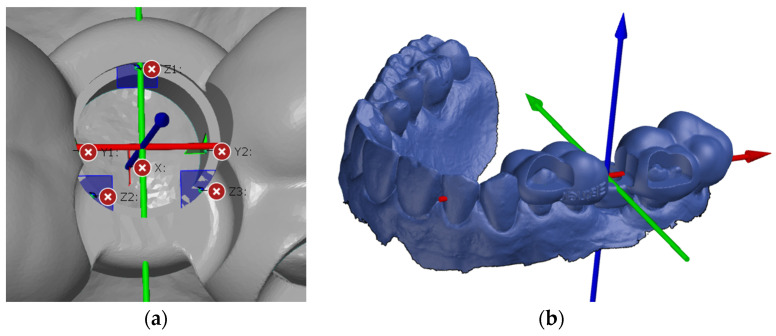
(**a**) Creation of the coordinate system; (**b**) conversion of the actual mesh to a CAD mesh.

In preparation for the first step, we imported the STL file of the planned template position to create a coordinate system. The software allows the three axes of the coordinate system to be defined individually using a “3-2-1 alignment”. The origin of the coordinate system should always be in the center of the cylinder of the planned implant position (Figure 3a). To ensure that the results could be compared, axes were created according to the same principle for all patient cases. The X-axis describes the direction from mesial to distal (i.e., positive values are distal, and negative values are mesial to the origin of the coordinate system), the Y-axis describes the direction from the vestibular to the oral site, and the Z-axis describes the direction from the apical to the occlusal site.

The current actual mesh was then converted into the CAD mesh, i.e., the target file. The change is clearly visible in the software, as actual files are displayed in grey and CAD files in blue (Figure 3b). The defined coordinate system was saved as the initial alignment of the CAD file. The scan with the template in the patient’s mouth could then be added to the component and imported as a new actual mesh.

**Figure 4:**
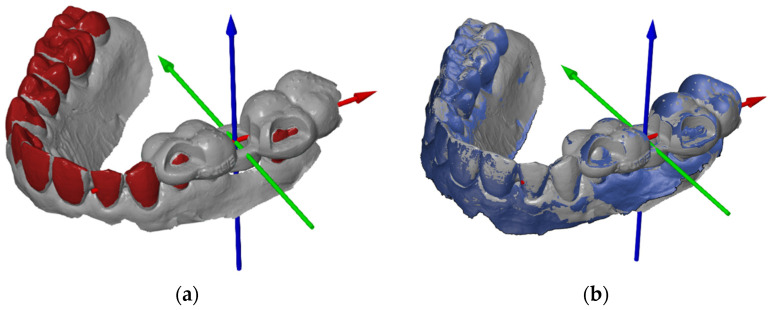
(**a**) Three-point-alignment; (**b**) matched actual and CAD mesh.

To achieve the most accurate possible overlay of the nominal and actual files, both an initial alignment and a main alignment were defined. Importantly, only the structures that remained the same in both files (in particular, the teeth) were superimposed, whereas the objects to be examined (the templates) were not superimposed under any circumstances. Otherwise, the actual deviation at the origin of the coordinate system would be incorrectly approximated or reduced. The initial alignment took place via “3-point alignment” (Figure 4a). The same points were marked on the teeth in both the actual and target files. Care was taken to ensure that the three points were distributed over the existing tooth arch width as evenly as possible. The necessary fine adjustments were carried out using the so-called “local best fit” as the main alignment. It was particularly important to leave out the template and only mark the teeth or, in the upper jaw, parts of the palate.

### 2.5. Construction of Virtual Geometries

**Figure 5:**
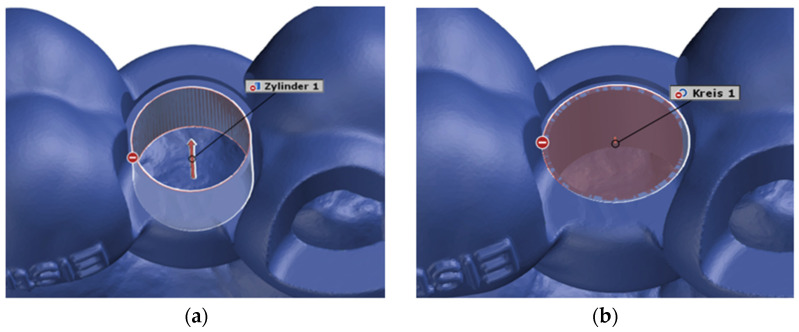
(**a**) CAD cylinder; (**b**) CAD circle.

**Figure 6:**
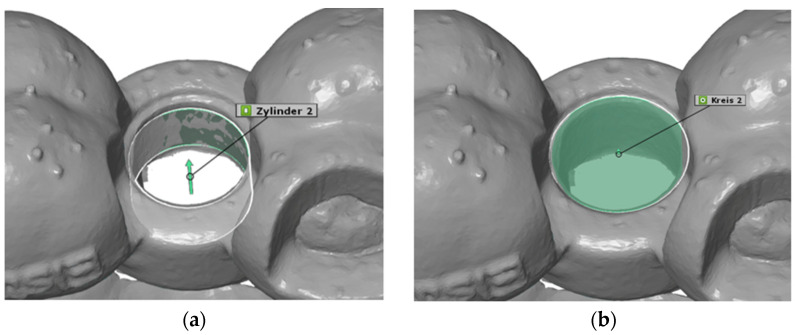
(**a**) Actual cylinder; (**b**) actual circle.

After superimposing the scans in this way, the starting conditions were created to begin the actual distance measurement. The deviation of the actual mesh from the CAD mesh was determined at the center of the opening of the surgical template for the implant drill holes, i.e., at the origin of the coordinate system. The underlying idea was to construct a virtual cylinder and circle at this opening of the surgical template in both the actual mesh and the CAD mesh. These two geometries were constructed as a fitting cylinder and a fitting circle via the “Gaussian best-fit” method (Figure 5 and Figure 6).

### 2.6. Measurement of the 3D Deviation

**Figure 7:**
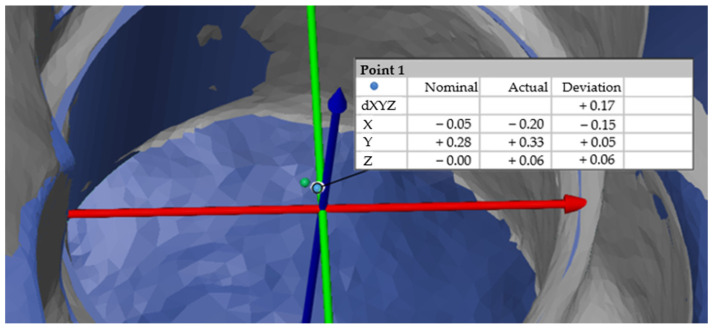
Distance measurement from the actual intersection point (green) to the nominal intersection point (blue).

Individual intersection points of the actual and target elements were then constructed (Figure 7). The individual deviations in the X, Y and Z axes were also determined, as was the deviation dXYZ, which corresponded to the Euclidean distance and therefore the straight-line distance between two points in a space [25]. The formula for calculating the Euclidean distance dXYZ in a three-dimensional space between the points $A\;\left(X_{1},\;Y_{1},\;Z_{1}\right)$ and $B\;\left(X_{2},\;Y_{2},\;Z_{2}\right)$ can be calculated as follows:

$$dXYZ=\sqrt{{\left(x_{2}-x_{1}\right)}^{2}+{\left(y_{2}-y_{1}\right)}^{2}+{\left(z_{2}-z_{1}\right)}^{2}}$$

#### Statistical Analysis

The data were analyzed using SPSS, version 25 software (IBM, Armonk, NY, USA).

As the long and short templates to be compared were each used on the same patient, the data collected in this study were from dependent samples. Patient-specific characteristics, such as anatomical structures and salivary flow, were constant for both measurements. By using identical patients as a basis, the pairing of the measurements was achieved, thus enabling direct comparisons.

The statistical analysis of the results was carried out via the t test for paired samples and the Wilcoxon signed-rank test. To ensure reliability in deviation measurements, the evaluation of the intraoral scans in “ZEISS INSPECT Optical 3D” was performed three times for each case by the same person. The three trials were conducted at different times, and each time the deviations from the planned position in the X, Y and Z directions and the vector dXYZ were determined.

## 3. Results

A total of 21 patients (male *n* = 15, female *n* = 6) with a total of 25 implants were analyzed in the study. Of the 25 implantations examined, the short templates resulted in a smaller deviation from the planned position by 20 times (80%). In contrast, the long templates showed a smaller deviation in only five cases. During the study, the short guide was actually used 15 times (60%), and the long guide was used 10 times (40%). Thus, the correct template was used 16 times (64%), and the less accurate template was used 9 times (36%).

**Figure 8:**
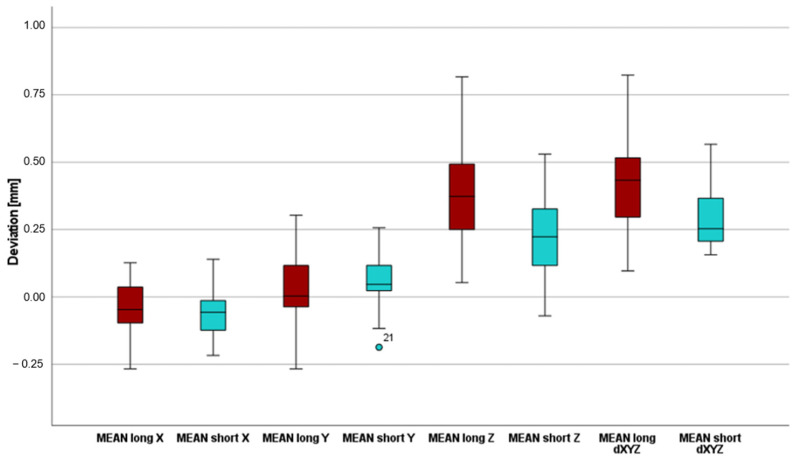
Boxplot as an overview of the various deviations on X-, Y-, Z- and dXYZ-axes.

The following tables provide an overview of the data collected in terms of the primary outcome. Table 1 shows that the deviations in the X direction, i.e., in the mesial–distal direction, were relatively similar, with average deviations of −0.0404 mm for the long templates and −0.0529 mm for the short templates (Figure 8). The negative sign in this study indicated that both template types are, on average, mesial to the planned position.

Table 2 shows the deviation in the vestibulo-oral direction (Y-direction), whereas the two average values differ by 0.046 mm (Figure 8). The long templates are, on average, 0.0101 mm farther apart orally, whereas the short templates are, on average, 0.0561 mm farther apart orally.

The largest deviations clearly occurred in the Z-axis (Table 3), i.e., from apical to occlusal. The mean deviations of 0.4007 mm for the long templates and 0.2275 mm for the short templates resulted in a difference of 0.1732 mm in this axis in favor of the short templates (Figure 8).

Regarding the deviations using the Euclidean distance dXYZ in three-dimensional space (as shown in Table 4), the total average deviation of the long templates is 0.4360 mm, and that of the short templates is 0.2953 mm. A clear difference of 0.1407 mm can therefore be recognized here (Figure 8). The results of the three measurement repetitions per case show a high level of agreement on the dXYZ axis. The average deviation from the mean value is 0.0376 mm for the short guides and 0.0380 mm for the long guides. Furthermore, the intra-rater reliability of repeated measurements was assessed using the intraclass correlation coefficient (ICC), based on a two-way mixed-effects model with absolute agreement and average measures (ICC). For the long drill guides, the ICC of the vector dXYZ was 0.977, indicating excellent reliability. For the short drill guides, the ICC for dXYZ was 0.929, also reflecting excellent consistency across repeated measurements by the same examiner [26].

**Figure 9:**
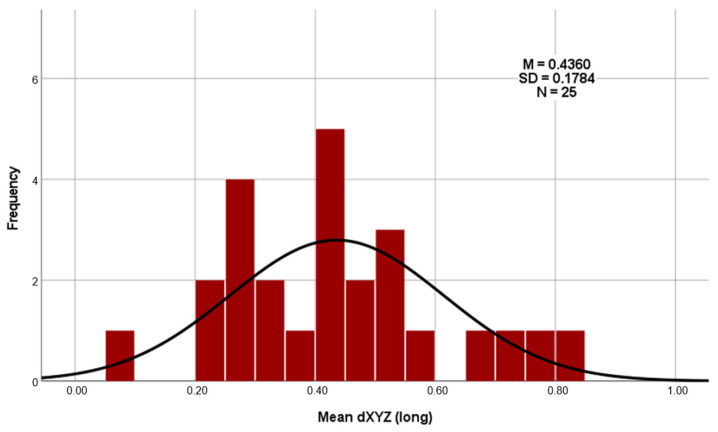
Histogram as an overview of the average deviation of the long templates.

**Figure 10:**
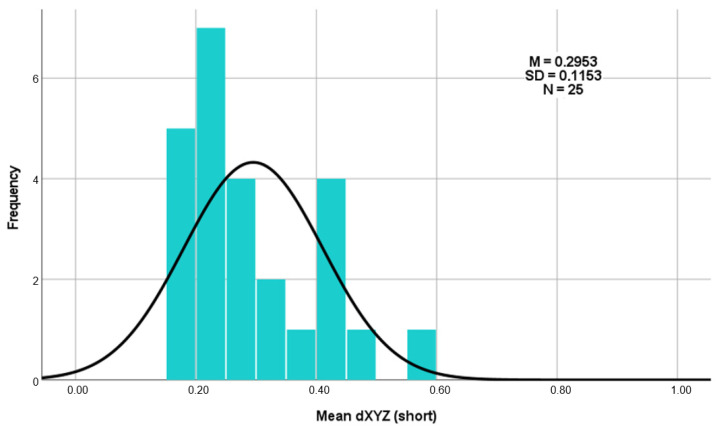
Histogram as an overview of the average deviation of the short templates.

The average deviation of the long templates ranged from a minimum of 0.10 mm to a maximum of 0.82 mm (Figure 9). For the short templates, the minimum deviation was 0.16 mm, and the maximum deviation was 0.57 mm (Figure 10).

On the basis of these average values of the Euclidean distances, a paired-samples t test was also performed. The null hypothesis of this t test was formulated as follows.

Null hypothesis (H_0): “The three-dimensional accuracy of the long and short templates is the same.”

The t test carried out (t (24) = 3.546; *p* = 0.002; d = 0.709) again shows that the short templates (M = 0.2953; SD = 0.11525) deviate significantly less from the planned template position than the long templates do (M = 0.4360; SD = 0.17841). As the *p* value (*p* = 0.002) is well below the significance level of 0.05 (*p* < 0.05), the null hypothesis must be rejected. This shows that there is a significant difference between the three-dimensional accuracies of the long and short templates in favor of the short templates.

The calculation of the effect size for Cohen’s d results in $d=\frac{\left|T\right|}{\sqrt{N}}=\frac{\left|-3.546\right|}{\sqrt{25}}=0.709$. According to the publication “A power primer” by Jacob Cohen from 1992, this effect size can be classified as between medium (0.50) and large (0.80) [27]. This proven discrepancy between the average deviations from the planned implant position is illustrated in the histograms (Figure 9 and Figure 10).

The 95% confidence interval of the T test indicates that there is a 95% probability that the true mean difference is between 0.05879 and 0.22255. This indicates a significant difference, as the value zero is not included in this interval.

The *p* value of 0.003 in the Wilcoxon test indicates a significant difference between the mean deviations of the short and long templates. It can therefore be assumed with high probability that the average values differ systematically, and that this difference does not just occur by chance. A post-hoc power analysis was conducted using G*Power, version 3.1 software (Heinrich Heine University, Düsseldorf, Germany). Given a two-tailed paired-samples t-test, a sample size of *n* = 25, an observed effect size dz = 0.709, and a significance level of α = 0.05, the achieved power was 0.925. These results indicate that the study had sufficient statistical power to detect effects of the observed magnitude [28].

Regarding secondary outcomes, the parameters under which fully guided implant surgery is still practicable were investigated. For this purpose, several examination parameters were recorded as clinical parameters, as described above.

In the cases analyzed, fully guided implant placement could be performed in 24 cases (from *n* = 25 implant insertions) (Table 5). In one patient, this implant placement did not work out; an implant was placed in region 16 in an edentulous opposing jaw. With a restricted mouth opening of 34 mm, the configurations for the guided surgery were as follows: sleeve position of 6 mm, drill tray configuration of +1 mm and drill length of 34 mm (total length of 40 mm and guided length of 24 mm).

In the molar region (lower jaw, here region 46), fully guided surgery is still achievable with a mouth opening of 40 mm if the maximum length and height are required for all configurations (sleeve position of 6 mm, drill tray configuration of +3 mm, and guided drill length of 24 mm). In the premolar region (lower jaw, here region 34), with the same maximum length and height configuration, a mouth opening of 32 mm was sufficient to perform the surgery in a fully guided manner.

Guided surgery in the molar region (and therefore also at any other implant position according to Spee’s curve) was performed without complications (even with the maximum height configuration) in patients with a mouth opening of ≥50 mm (Table 5).

Additionally, whether preferences for a certain type of template design could be identified depending on the position of the implant placed by the surgeon was investigated.

In all four quadrants, the short template was the one with the better fit, while surgery was favored in approximately 80% of the cases (first quadrant, 83%; second quadrant, 80%; third quadrant, 75%; fourth quadrant, 80%).

## 4. Discussion

The introduction of CAD/CAM and navigation-assisted technologies into surgical operations has significantly improved precision and efficiency in medicine, and the use of static computer-aided surgery (sCAIS) not only plays an important role in training at universities [29], but is also increasingly becoming part of everyday practice for surgical dentists and their patients. CAD/CAM makes it possible to create precise 3D models of patient structures, which are then used to plan and manufacture customized features. This leads to more individualized treatment and a reduction in complications. Navigation technology, on the other hand, provides real-time visualization during surgery and allows the surgeon to monitor the exact position of meshes or implants in the patient’s body. This is not only used in implantology, but also in other fields of surgery, where the goals are the same and the same difficulties have to be managed, namely, the highest possible precision in implementation. This also allows surgeons to perform guided bone regeneration in a virtually navigated manner. With the help of 3D data sets, meshes can be pre-formed and manufactured individually for the patient to promote prosthetically guided bone reconstruction, and to achieve optimal implant placement and prosthetic finalization (reverse guided bone regeneration). A study aimed to examine the accuracy of a digital protocol for such reverse guided bone regeneration with CAD/CAM 3D meshes. Converting the computer tomography datasets from before and after guided bone regeneration into 3D models and aligning them digitally, the authors compared the actual position of the mesh to the virtual position to assess the accuracy in 16 patients with horziontal and vertical bone defects. They revealed an overall mean discrepancy between the virtual and actual positions of the mesh of 0.487 ± 0.218 mm. However, no statistically significant difference was observed when comparing this to a predefined minimum tolerance (*p* = 0.06), thus this digital protocol seems to be able to achieve good accuracy [30].

For the practitioner, the detailed planning and guided surgery of implants provides certainty in terms of predictability and avoidance of complications, particularly damage to neighboring structures [19]. For the patient, a result can ideally be achieved that corresponds to the previous initial situation with their own teeth. This approach might also save the patient a certain amount of time during the operation, which, in turn, leads to less postoperative pain or swelling. However, the literature has reported different findings. One study compared different surgical approaches and revealed that sCAIS took the longest time (89.70 ± 45.75 min), followed by dCAIS (dynamic computer-aided surgery) (70.95 ± 42.48 min) and the FH protocol (70.30 ± 47.08 min) [31]. However, the patients in that study experienced significantly less pain with sCAIS (pain score 0.77 ± 1.85) than with the FH approach after one week (pain score 1.40 ± 2.43), but the difference was not significant.

A systematic review revealed that the surgical process itself derives a time advantage of 18–23 min when using sCAIS, whereas other studies showed no time advantages. However, the authors concluded that when the whole treatment workflow is considered (diagnostics, planning, and surgery), sCAIS seems to lead to advantages over non-sCAIS protocols in terms of time and effort [32]. Therefore, even if full guidance with the sCAIS approach is not possible, 3D planning is still recommended for the predictability of the implant outcome. While in our evaluation, the fully guided approach worked in 24 out of 25 cases, in the remaining case, 3D planning was also carried out. Nevertheless, the proportion of sCAIS implant insertions and operations in relation to total implants inserted is still rather low, although this approach is also clearly superior to conventional treatment in terms of accuracy and complications [2]. One review examined different parameters with respect to the deviation of implants and different surgical protocols. In terms of the angular deviation of the implant, values were generally greater in FH (6.90 ± 4.40° to 9.92 ± 6.01°) than in computer-aided (2.20 ± 1.10° to 5.95 ± 0.87°) approaches [3]. The 3D bodily deviations exhibited less drastic values between the FH (coronal—1.25 ± 0.62 to 2.77 ± 1.54 mm; apical—2.10 ± 1.00 to 2.91 ± 1.52 mm) and computer-aided (coronal—0.54 ± 0.33 to 2.34 ± 1.01 mm; apical—0.90 ± 0.43 to 2.53 ± 1.11 mm) approaches. With respect to the depth deviation of the implants from the actual planning, only two studies were described that took this approach; however, these studies were unable to show any significant value changes between FH (0.50 ± 0.09 mm) and sCAIS (0.43 ± 0.09 mm), despite the fact that the accuracy showed consistently superior outcomes in sCAIS to FH. This finding is consistent with our results, as we also saw the greatest axis deviation in the Z-axis, i.e., the depth to the originally planned position. However, this deviation was less pronounced with the short templates than with the long templates.

Interestingly, however, sCAIS is currently increasingly evolving into dCAIS (dynamic computer-aided surgery) [33], i.e., away from implantation via a drilling template, a drilling sleeve and, correspondingly, extended drills [34]. One reason for this evolution may be the expansion of the possibilities of new technologies; however, the problems of implementing the sCAIS approach and its features are certainly also a reason. The deviation of the actual implant position from the planned position is a frequently discussed factor within sCAIS and can be expected [35,36,37]. This also corresponds to our results, as we saw a deviation in every X-, Y- and Z-axis from the planned position in both templates. However, clinical acceptance, even with differing angular deviation, is always given [38]. There are many reasons for this deviation, e.g., the material of the template and thus minor errors during manufacture, the surgical approach (fully guided with guided implant insertion or half-guided), or simple human errors [39]. More problems for the use of sCAIS can manifest in different ways: splint fractures, drill access, the insufficient fit of the template or slipping, and thus non-usability (splint adaptation and retention), as well as restricted mouth opening, or a good template fit but slippage of the template during drilling, as a result of which the practitioner or assistant cannot hold it completely in the final position [40]. All of these phenomena ultimately lead to the switch to FH. Additionally, unforeseen events can occur intraoperatively (e.g., the patient simply does not tolerate the template), which the surgeon must be able to address. However, to our knowledge, no study has yet been carried out on the design of the template in vivo. In fact, in terms of deviation between the planned and actual implant, there are in vitro studies that have evaluated different designs of the printed templates. A full-arch-supported guide, three different tooth-supported short surgical guides and a full-arch-supported guide with a crossbar were evaluated. They concluded that all templates had a clinical acceptable level of deviation; however, the accuracy for short templates in a single-edentulous site was higher, whereas the full-arch template with a crossbar had higher accuracy with two or more implants [41]. In another in vitro study, there were 375 implants inserted into 85 study models. In this case, 3D-printed surgical guides were either designed to be supported by all the teeth or by four teeth, three teeth or two teeth. The study models included three single-tooth gap situations. They concluded that the accuracy of surgical guides was significantly affected by the number and also the type of teeth used for support, whereas four-tooth support provides equal accuracy compared to full-arch guides [38]. This correlates to our study design, because we compared the fit of a full-arch guide to a short one with at least four-tooth support with a lower deviation in the evaluated 3D axes. We might suppose that the angular deviation of the actual implant differs from the planned position in both template designs, but we did not evaluate this in our study because we wanted to focus on the fit in the patients’ mouth. Moreover, as stated in the literature, the angulation degrees for either full-arch or short guides are clinically acceptable. But this could be a subject of evaluation for further studies. Still, the question remains as to what effects these deviations might have on the longevity of implants, or in what range are these deviations still clinically acceptable. To answer this question, we have to refer to studies that not only examined the accuracy of different procedures or surgical guides, but also the implant success rate. A prospective study found that there were non-significant deviations between conventional (C) and sCAIS surgical guides, which is quite in line with the findings of previous literature (coronal deviation conventional 1.52 mm to sCAIS 1.04 mm, apical from 1.67 mm to 1.46 mm and an overall angle deviation from 2.87° to 3.64°); however, the implant success rate within this population at 1 year was 90% in the conventional group vs. 100% in the sCAIS group. However, they concluded that it was not the guide that influenced the success rate, but statistically relevant variables such as implant length, drill access and type of flap design [40]. Thus, we might conclude that the deviations have no impact on the implant success rate. In view of prosthetic failures, there are no correlations with implant angulation, as the multi-unit abutments can even compensate angulations of up to 80° in some cases [42,43]. This means that the described angular deviations are within the absolutely clinically acceptable range.

However, if we look at the possible increased bone resorption rate with angulated implants, there is a retrospective study that evaluated the correlation between the magnitudes of multidirectional implant angulations and peri-implant crestal bone loss. It included 288 patients with 506 dental implants, and the mean follow-up duration after laoding was 5.1 years [44]. The authors observed a signifincant deviation of peri-implant bone loss of 0.10 ± 0.39 mm in the axial implants and 0.22 ± 0.48 mm in the nonaxial implants, and concluded that nonaxially positioned implants exhibited greater bone loss compared with axially positioned implants. Thus, a careful consideration of implant angulation is crucial in minimizing peri-implant bone loss. However, it was not explicitly reported here how the angular deviation came about, or what influence the CAD/CAM accuracy had. In addition, there are many other factors that influence peri-implant bone loss, such as the thickness of the gingiva [45] or whether soft tissue augmentation [46] has taken place, and it is known that some marginal bone loss can occur because of several other factors [47].

Of course, few studies have focused on a patient’s restricted mouth opening in advance of the planned operation; however, we can only use known values from the literature to make the decision for or against sCAIS. One study investigated the maximum degree of mouth opening required for sCAIS fully guided implant surgery. The vertical dimension of the drills in the handpiece ranged from 30.0 to 49.5 mm, and the height of the surgical guide sleeve on top ranged from 3.2 to 13.5 mm, which requires a total vertical space of 30.0 to 58.5 mm, regardless of which system is used [21]. In our study, we observed the lower limit of the use of sCAIS for the mandible in the molar region with the maximum possible height (template + drill sleeve + drill tray + drill) at 40 mm mouth opening, and in the premolar region at 32 mm. In the molar region of the maxilla, on the other hand, we observed a lower limit of 34 mm. Anything with a mouth opening ≥50 mm did not pose a problem when using the sCAIS. Therefore, mouth opening should be included as a standard in the initial surgical preparation for decision-making during the surgical procedure, and as a possible factor for any complications.

The inadequate fit of a surgical guide can be improved either by use of different materials, i.e., different manufacturing processes, such as the printing process (different 3D printing processes), or via the assessment of intraoral information (digital vs. conventional) [48]. Templates that are 3D-printed are also used in the field of surgical traumatology. Here, a systematic review found that the accuracy of such patient-specific surgical guides averages around 2–3° due to fitting; however, their use in complex cases appears to make sense [49]. That said, even in the field of surgical orthopedics, there are currently not enough studies describing the accuracy in all planes, which is in line with the problems encountered in guided implantology that are addressed in this study. In this field, the inadequate fit of a surgical guide can manifest as template movement due to the excessively large contact surface, e.g., over the entire dental arch, because there are potentially more teeth interfering with the length of the template. The presence of adjacent teeth to support the template or the surgeon’s level of experience could therefore also be factors that influence the outcome [50]. Regarding the use of the full guided approach, there were no correlations between the accuracy of implant position and the level of experience of the surgeon. The inexperienced ones performed comparably [51]. Another clinical problem with a longer template is that the surgeon or assistant cannot hold the template in situ during surgery, especially when drilling the implant. If the surgeon is right-handed, it is difficult for him or her to hold the long template with one hand over the floor of the mouth or tongue in the final position during implant placement in the third quadrant, for example. A remedy here could be to print the template with a narrower offset, but then it cannot be removed so easily and quickly to check the position when checking the drill holes. The aim is to shorten the operation time with sCAIS, which has been shown to be possible in 7.58–10.5 min [52,53].

All these findings form the basis of this in vivo study, namely, changing the design of the template and favoring shorter ones, which has already been shown in vitro [38,41]. In a 2017 study, 13 full-arch guides were analyzed with respect to the 3D deviation of the implant position. Here, the deviation was greatest in the Z-axis direction, which is consistent with our results. However, the mean deviation in the dXYZ-axis (0.864 mm) was greater than that of our long guides, and even greater than that of our short guides. The values of the long template can be explained by the fact that we measured them in triplicate, but the short template values could be related to the design. Other reasons for error are also discussed here, including X-ray imaging; template fabrication; inaccuracies from positioning and template movement; and patient-related limitations (e.g., maximum opening of the mouth). The authors called for further research in terms of evaluating the deviation between the virtually planned position and the surgical template as the main factor [25]. Moreover, the fact that patients have increasing zirconia crowns could be a problem when using sCAIS. The accuracy of the superimposition of the images is influenced by the number of zirconia crowns, with an increased number reducing the superimposition accuracy within the planning process of the implant regarding the template [54]. This fact was not evaluated; however, we saw no interferences whatsoever in the superimpositions performed in every plan, which were only carried out by an experienced surgeon. Thus, in this study, all templates used were fabricated via an intraoral scan and a DLP printing process, and the fit of the short or long template was compared with the previously digitally planned fit based on the implant position in the patient’s mouth. The 3D fit of the short template in the patient’s mouth deviated significantly less from the planned implant position. Additionally, the short template was actually used by the surgeon for the operation in 60% of cases in terms of handling. However, further studies will be needed to compare sCAIS and maybe also dCAIS in vitro with regard to the angular deviation of the implants, even if the angulation has no deep impact on the longevity of the implants, as long as enough bone and soft tissue is present around the implants [55]. Moreover, in vivo studies are also needed regarding short template designs and the angular deviation to evaluate the accuracy.

However, there are a few shortcomings of the study that need to be mentioned and discussed. First, the actual position of the implant in relation to the initial planning situation compared with the templates used was not considered here. This could have been done via a cone beam computed tomography (CBCT) scan or by evaluating the position via scan bodies; however, since deviations in sCAIS have been sufficiently described, this factor should not have played a decisive role in the success of this evaluation method. Additionally, only a small number of patients and not every position in every quadrant could be mapped in a representative manner, but this also enables follow-up studies, whereby the correlation of mouth opening with the parameters would also be more meaningful. Moreover, a sample size calculation for the critical evaluation of the results should be performed in advance for further studies. However, to reduce bias, each case was measured three times (by one examiner), and all operations were performed by one surgeon only.

## 5. Conclusions

The design and fit of the template play an important role in the feasibility of a planned sCAIS approach in patients’ mouths. Templates with at least a four-tooth-supported design in terms of single-gap implant restorations are preferred. Even if the depth deviation is greater than in other axes, there was less deviation in the short guide template. The position of the implant in relation to the mouth opening also plays an important role in the feasibility of sCAIS. However, these results definitely need to be validated in follow-up studies with larger cohorts.

## Figures and Tables

**Table 1:** Deviation in the X direction (mesial–distal). The values marked in bold each represent the mean of the 3 evaluations on the X-axis (mesial (–) distal (+)).

	*N*	Minimum (mm)	Maximum (mm)	Mean (mm)	Std. Deviation
T1 long X	25	−0.29	0.14	−0.368	0.11550
T2 long X	25	−0.28	0.14	−0.0452	0.10767
T3 long X	25	−0.23	0.11	−0.0392	0.08426
**Mean long X**	25	−0.27	0.13	**−0.0404**	0.09939
T1 short X	25	−0.25	0.16	−0.0480	0.09161
T2 short X	25	−0.18	0.15	−0.0520	0.08495
T3 short X	25	−0.22	0.13	−0.0588	0.10465
**Mean short X**	25	−0.22	0.14	**−0.0529**	0.08680

**Table 2:** Deviation in the Y direction (vestibulo-oral). The values marked in bold each represent the mean of the 3 evaluations on the Y-axis (vestibulo (–) oral (+)).

	*N*	Minimum (mm)	Maximum (mm)	Mean (mm)	Std. Deviation
T1 long Y	25	−0.29	0.36	0.0180	0.12748
T2 long Y	25	−0.29	0.33	0.0064	0.12832
T3 long Y	25	−0.23	0.22	0.0060	0.11587
**Mean long Y**	25	−0.27	0.30	**0.0101**	0.12170
T1 short Y	25	−0.18	0.29	0.0692	0.11543
T2 short Y	25	−0.19	0.28	0.0580	0.09721
T3 short Y	25	−0.19	0.20	0.0412	0.09391
**Mean short Y**	25	−0.19	0.26	**0.0561**	0.09901

**Table 3:** Deviation in the Z direction (apical–occlusal). The values marked in bold each represent the mean of the 3 evaluations on the Z-axis ((apical (–) occlusal (+)).

	*N*	Minimum (mm)	Maximum (mm)	Mean (mm)	Std. Deviation
T1 long Z	25	0.02	0.83	0.3984	0.19237
T2 long Z	25	0.10	0.77	0.4052	0.18058
T3 long Z	25	0.04	0.85	0.3984	0.19570
**Mean long Z**	25	0.05	0.82	**0.4007**	0.18591
T1 short Z	25	−0.21	0.44	0.2068	0.16683
T2 short Z	25	−0.06	0.60	0.2360	0.16837
T3 short Z	25	0.05	0.67	0.2396	0.15263
**Mean short Z**	25	−0.07	0.53	**0.2275**	0.15220

**Table 4:** Deviation in XYZ direction (Euclidean distance). The values marked in bold each represent the mean of the 3 evaluations of the vector dXYZ.

	*N*	Minimum (mm)	Maximum (mm)	Mean (mm)	Std. Deviation
T1 long dXYZ	25	0.03	0.84	0.4368	0.18663
T2 long dXYZ	25	0.15	0.78	0.4416	0.17380
T3 long dXYZ	25	0.11	0.85	0.4296	0.18687
**Mean long dXYZ**	25	0.10	0.82	**0.4360**	0.17841
T1 short dXYZ	25	0.13	0.47	0.2932	0.10816
T2 short dXYZ	25	0.10	0.62	0.2944	0.13629
T3 short dXYZ	25	0.16	0.68	0.2984	0.12439
**Mean short dXYZ**	25	0.16	0.57	**0.2953**	0.11525

**Table 5:** Overview of important parameters of the implantations performed and demographic information of the patients.

Patient ID	Implant Position	Number of Supported Teeth in Long Guide	Number of Supported Teeth in Short Guide	Age (Years)	Sex	Interincisal Distance (mm)	Feasibility of Fully Guided	Template Used	Lower Deviation dXYZ
1	21	13	4	45	female	41	yes	short	short
2	46	13	4	58	male	40	yes	short	long
3	34	11	4	59	male	32	yes	long	long
4	34	11	4	71	male	41	yes	short	short
5	46	13	4	54	male	54	yes	short	short
6	16	13	4	51	male	42	yes	short	short
7	21	10	4	84	male	50	yes	short	long
8	24	15	4	49	male	49	yes	short	short
9	16	14	4	50	male	52	yes	short	short
10	16	11	4	73	male	34	no	long	long
11	24	12	4	84	female	45	yes	long	long
12	35	13	4	50	male	46	yes	long	short
13	14	13	4	80	female	38	yes	short	short
14	46	13	4	51	male	42	yes	short	short
15	36	13	4	65	male	58	yes	long	short
16	22	13	4	57	female	48	yes	long	short
17	26	12	4	80	female	40	yes	short	short
18a	24	11	4	70	male	40	yes	short	short
18b	25	11	4	70	male	40	yes	short	short
19a	45	12	4	51	female	48	yes	long	short
19b	47	12	4	51	female	48	yes	long	short
20a	24	11	4	59	male	56	yes	long	short
20b	26	11	4	59	male	56	yes	long	short
21a	15	11	4	71	male	52	yes	short	short
21b	16	11	4	71	male	52	yes	short	short

## Data Availability

The datasets used and/or analyzed during the current study are available from the corresponding author on reasonable request.
